# A Floating Capsule Electrochemical System for In Situ and Multichannel Ion-Selective Sensing

**DOI:** 10.3390/bios13100914

**Published:** 2023-10-05

**Authors:** Jie Yang, Ao Ding, Jia-Le Zhou, Bing-Yong Yan, Zhen Gu, Hui-Feng Wang

**Affiliations:** 1 Key Laboratory of Smart Manufacturing in Energy Chemical Process Ministry of Education, East China University of Science and Technology, Shanghai 200237, China; 2 School of Chemistry and Molecular Engineering, East China University of Science and Technology, Shanghai 200237, China

**Keywords:** capsule sensor, solid-contact ion-selective electrode, in situ monitoring, multichannel, wireless sensor network, cell culture

## Abstract

Free-floating electrochemical sensors are promising for in situ bioprocess monitoring with the advantages of movability, a lowered risk of contamination, and a simplified structure of the bioreactor. Although floating sensors were developed for the measurement of physical and chemical indicators such as temperature, velocity of flow, pH, and dissolved oxygen, it is the lack of available electrochemical sensors for the determination of the inorganic ions in bioreactors that has a significant influence on cell culture. In this study, a capsule-shaped electrochemical system (iCapsuleEC) is developed to monitor ions including K^+^, NH_4_^+^, Na^+^, Ca^2+^, and Mg^2+^ based on solid-contact ion-selective electrodes (SC-ISEs). It consists of a disposable electrochemical sensor and signal-processing device with features including multichannel measurement, self-calibration, and wireless data transmission. The capacities of the iCapsuleEC were demonstrated not only for in situ measurement of ion concentrations but also for the optimization of the sensing electrodes. We also explored the possibility of the system for use in detection in simulated cell culture media.

## 1. Introduction

Recently, the rapid development of cell therapy technologies such as stem cell therapy and chimeric antigen receptor T (CAR-T) cell therapy raises the requirements for the development of bioreactors for large-scale cell culture [1,2,3]. Among these, online sensors are essential components in bioreactors for the monitoring of a considerable number of variables such as pH, dissolved oxygen, cell density, and metabolite concentrations, promoting the optimization of culture parameters and enabling feedback control of the bioprocess [4]. The culture medium provides essential nutrients for cell growth in bioreactors, in which inorganic salts including Na^+^, K^+^, Ca^2+^, and Mg^2+^ play a crucial role in maintaining the osmotic pressure balance of the cell culture solution and participating in cellular metabolism [5]. On the other hand, the accumulation of NH_4_^+^ produced by cell metabolism can disrupt the substrate metabolism balance of cells and affect cell growth [6,7,8]. Therefore, it is of great significance to effectively monitor the concentration variations of inorganic ions in bioreactors for both cell culture studies and the scale-up of the culture process.

Free-floating wireless sensors are an emerging technique for performing measurements without the need for a physical connection to a bioreactor [9,10]. A capsule-shaped bioprocessing online device (BPod) platform was developed to monitor dissolved oxygen (DO) levels in real time within a bioreactor by using a disposable electrochemical sensor [11]. It can float inside the bioreactor and wirelessly transmit the measurement data to a smartphone. The advantages of wireless sensors are obvious, including (1) lowering the complexity of bioreactors; (2) lowering the risk of contamination; and (3) performing measurements at different sites in the bioreactors. However, most of the studies of floating electrochemical sensors are focused on the measurement of pH and DO. More efforts are still need to develop online sensors for inorganic ions in cell culture applications. To develop a floating electrochemical sensor for bioreactors, the challenges include (1) the integration of the sensor and device; (2) multichannel sensing for different ions; (3) long-term usage inside the bioreactor; and (4) the complexity of the cell culture media.

Potentiometric sensors have the advantages of fast response time, wide measurement range, and low production cost; they are used in the industry to monitor pH [12], DO [13], carbon dioxide (CO_2_) [4], glucose, and glutamate [14]. Potentiometric solid-contact ion-selective electrodes (SC-ISEs), which replace the internal reference solution with a solid-contact membrane, have the advantages of simple structure, easy fabrication, easy miniaturization, timeliness, and reliability; thus, they are widely applied in submersible probes [15], lab on a chip [16], soil analysis [17,18], biomedicine [19,20], wearable devices [21,22,23], etc. In particular, the advantages of high sensitivity and fast response make it possible to monitor the dynamic changes in ion concentration in a bioreactor. At present, poly(3,4-ethylenedioxythiophene) polystyrene sulfonate (PEDOT: PSS) is one of the most promising candidate materials for the solid-contact layer for ion–electron transducers due to its excellent ion–electron conductivity and low sensitivity to O_2_ and CO_2_ [24]. However, due to the presence of hydrophilic PSS, a water layer forms between the ion-selective membrane and the solid-contact layer, which contributes to potential drift and decreases the lifetime of the electrodes [25,26]. Two kinds of two-dimensional MXene nanosheets are utilized as ion–electron transducers to fabricate solid-contact Ca^2+^ ion-selective electrodes [27]. MXene-coated electrodes exhibit fast response speed (less than 10 s), outstanding potentiometric performance, extraordinary long-term stability, and insensitivity to light, CO_2_, and O_2_, showing satisfactory prospects in conventional sensing applications.

In this study, we developed a multichannel integrated capsule electrochemical system called iCapsuleEC for the in situ and wireless detection of inorganic ions. It has the features of small size, multichannel measurement, long-term usage, and battery supply. To demonstrate the ability of the iCapsuleEC for electrode optimization and in situ detection, it was used to investigate the performance of a solid-contact layer based on Ti_3_C_2_ for the multichannel detection of K^+^, NH_4_^+^, Na^+^, Ca^2+^, and Mg^2+^. Then, the iCapsuleEC based on the optimized SC-ISEs was used to monitor the ion concentrations in a simulated cell culture media. 

## 2. Experimental Section

### 2.1. Chemicals and Materials

The list of chemicals and materials in the experiment is provided in Supporting Information S1.

### 2.2. Design of the iCapsuleEC System 

**Figure 1:**
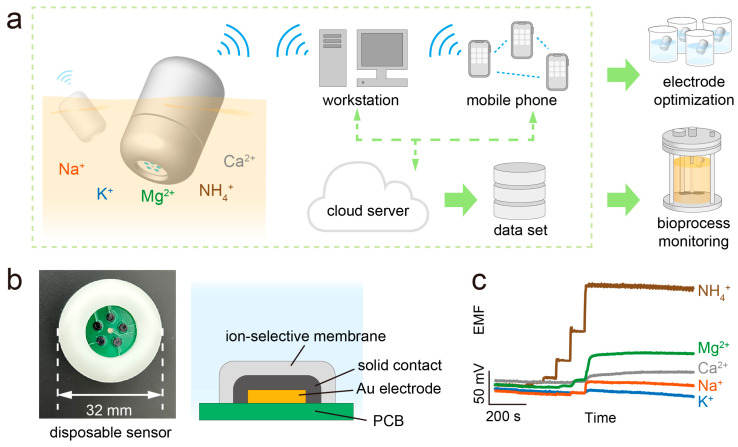
(**a**) Design and application of the iCapsuleEC platform with features of high integration and wireless data acquisition for electrochemical sensing. (**b**) The disposable sensor with integration of five solid-contact ion-selective electrodes and reference electrode. (**c**) Continuous recording of the EMF signal by the iCapsuleEC for online inorganic ions monitoring.

The iCapsuleEC system is a capsule-shaped sensor system that can float in a bioreactor for the in situ and wireless detection of inorganic ions (including K^+^, NH_4_^+^, Na^+^, Ca^2+^, and Mg^2+^) based on integrated SC-ISEs. It consists of a disposable electrochemical sensor (DES) and a signal-processing device (SPD). The BLE (Bluetooth low energy) technique is selected for wireless data transmission as it has the advantages of low power consumption, low cost, ease of connection, and compatibility with most computers or mobile phones. In addition, a wireless sensor network can be established with more than two SPDs by using a mesh network. Each SPD in the network performs both data acquisition and data routing. Therefore, the devices are able to send data to a terminal (computer or mobile phone) from a long distance, and the terminal can also collect data from multiple devices. The acquired data on the terminal can be uploaded to a cloud server to inform remote users and build data sets for further analysis (Figure 1a). Due to the high efficiency of the iCapsuleEC system for data collection and analysis, it can be used not only to optimize the design and material of the SC-ISEs but also to help monitor bioprocesses that are sensitive to the concentrations of inorganic ions. 

Since the target ions in different bioprocesses are not the same and the SC-ISEs may be contaminated or damaged during usage, the DES is designed as a module that is replaceable (Figure 1b). The SC-ISEs in the DES are fabricated on a printed circuit board (PCB) with five working electrodes (2 mm in diameter) and a Ag/AgCl reference electrode (2 mm in diameter) in the center. The inner layer of the DES is a gold electrode. Then, solid-contact and ion-selective membranes are formed on the gold electrode. 

During measurement, the concentration of target inorganic ions can be evaluated based on the electromotive force (EMF) measurement of the SC-ISEs (Figure 1c).

### 2.3. Hardware and Software 

**Figure 2:**
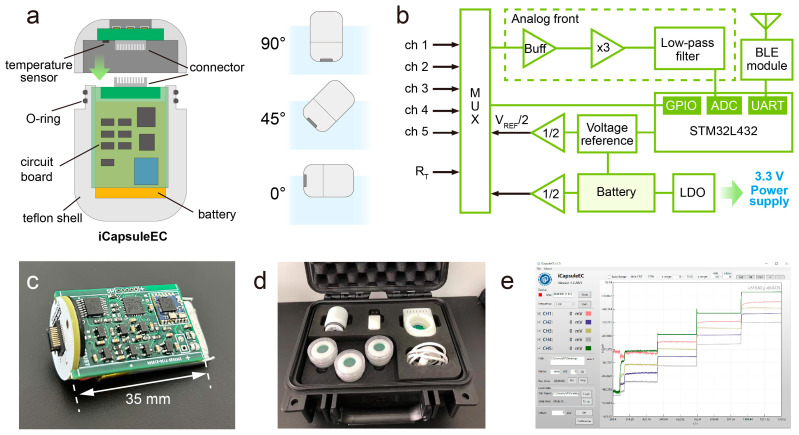
(**a**) The components of the iCapsuleEC. The gravity center of the device is optimized to achieve a 45° posture in water solution. (**b**) The schematic diagram of the circuit board. (**c**) A photograph of the signal−processing circuit board. (**d**) The portable package of the iCapsuleEC platform. (**e**) The software interface for data acquisition and visualization.

The detailed structure of the iCapsuleEC is shown in Figure 2a. The DES and the SPD both have PTFE-made shells; therefore, the two parts can be assembled to form a capsule. Board-to-board connectors are used as the electrical connection for the two parts. Two rubber O-rings are fixed at the interface of the two shells to ensure that the whole system is waterproof. The SC-ISEs are fabricated on the outside of the DES. Hence, the electrodes can be immersed in the solution. The angle between the long side of the capsule and the horizontal plane is optimized to 45° in practice by adjusting the center of gravity of the capsule, since the configuration of both large and small angles may lead to inadequate contact of the electrodes with the solution. For example, bubbles may easily stay on the electrodes with a configuration of 90°, while parts of the electrodes could be higher than the surface of the water with a configuration of 0°. The SPD part is an integration of the circuit board and the chargeable Li battery (120 mAh). It can work for over 20 h with a sampling interval of 1 s.

The circuit of the SPD can convert, acquire, process, and transmit the signals from the SC-ISEs. It includes a multiplexer (MUX), an analog front-end (AFE), a microcontroller (MCU), a voltage reference, a low-dropout regulator (LDO), and a BLE module (Figure 2b). A STM32L432 (STMicroelectronics Inc., Geneva, Switzerland) was selected as the MCU due to its high integration and low power consumption. It has an on-chip 12-bit analog-to-digital convertor (ADC), which can work in an oversampling mode to achieve 16-bit resolution. The multiplexer (ADG1408, Analog Devices Inc., Norwood, MA, USA) is used to switch the input for the AFE. For the calibration of signal measurement, the MUX connects the AFE with the signal at ground or half of the reference voltage (reference zero). Temperature can also be measured by connecting the AFE to a thermistor (RT). During a cycle of measurement, the MUX connects the signal channels (CH1 to CH5) to the AFE in sequence to acquire the potential of each working electrode. The AFE amplifies the input signal 3 times by using an operational amplifier (AD8605, Analog Devices Inc., Norwood, MA, USA) to increase the signal-to-noise ratio; then, a 2-pole low-pass Bessel filter is used to denoise the signal with a cut-off frequency of 10 Hz. The filtered signal is then fed into the on-chip ADC of the microcontroller. The final resolution for potential measurement is approximate 12 μV. The converted data in the MCU is transmitted to the Bluetooth module by using UART communication. To improve the stability of measurement, a reference voltage source (ADR5043B, Analog Devices Inc., Norwood, MA, USA) is used to provide 3 V standard voltage, and then a 1.5 V voltage is generated as the reference zero by amplifying the reference voltage with a gain of 0.5. The reference zero is connected to the reference electrode to enable measuring the negative voltage on the working electrode. The circuit is powered by a 3.3 V voltage source, which is obtained by regulating the voltage from a lithium-ion battery by a low-dropout regulator (TPS79333, Texas Instruments Inc., Dallas, TX, USA). The circuit board in the SPD is only 35 mm in length (Figure 2c), and the whole system can be put into a small suitcase for portable use (Figure 2d).

A computer software program with a user-friendly interface was developed based on the framework of the Windows presentation foundation through Visual Studio 2019 for online data acquisition, recording, and visualization of the signals from the SC-ISEs (Figure 2e). For data recording on a mobile phone, we also developed a WeChat app, which runs on both the iOS and Android systems.

### 2.4. Prepreparation of the PCB-Au/SC/ISE-Based Sensor 

The DES consists of five working electrodes (PCB-Au/SC/ISMs) and one reference electrode (Ag/AgCl electrode). The PCB-Au/PEDOT:PSS/ISEs were fabricated by drop-coating 5 µL of commercial PEDOT:PSS solution onto the working electrodes (2 mm in diameter) and drying at 80 °C for 2 h. After cooling to room temperature, 5 µL of each ion-selective membrane cocktail solution was drop-coated onto the PEDOT:PSS layer and air-dried overnight at room temperature to prepare the solid-contact K^+^, NH_4_^+^, Na^+^, Ca^2+^, and Mg^2+^-ISEs based on PEDOT:PSS. The PCB-Au/Ti_3_C_2_/ISEs were fabricated by drop-coating 5 µL of 5 mg/mL Ti_3_C_2_ solution onto each of the five working electrodes. The electrodes were air-dried at room temperature for 5 h to prepare the Ti_3_C_2_ layer. Then, drop-coating 5 µL of five membrane cocktail solutions on the Ti_3_C_2_ layer was performed, followed by air-drying overnight to prepare PCB-Au/Ti_3_C_2_/ISEs for K^+^, NH_4_^+^, Na^+^, Ca^2+^, and Mg^2+^. The compositions of ion-selective membrane (ISM) cocktails are listed in Table 1. The total mass of 500 mg of the membrane components was dissolved in 5 mL of THF to prepare the ISM cocktail solution. The prepared cocktails were sealed and stored at 4 °C. The reference electrode was fabricated by screen-printing silver ink (JY20, Julonghuina Co., Ltd., Shanghai, China) on the PCB-Au, which was then dried at 80 °C for 2 h. After cooling to room temperature, the reference electrode was immersed in NaClO solution for 1 h to obtain the Ag/AgCl electrode. To seal the sensor, the PCB was fixed in a 3D-printed cover. Polydimethylsiloxane (PDMS) was injected into the gap between the PCB and the cover, and the assembly was heated on a 70 °C heating plate for 1 h to solidify the PDMS and achieve a waterproof seal for the sensor. The chemicals and materials used above are listed in Supplementary Materials S1.

### 2.5. Testing for Potential Measurement 

To test the accuracy and consistency of each channel in the SPD, all channels (CH1 to CH5) were connected to a reference voltage ranging from −400 mV to 400 mV with a step of 100 mV generated by a functional generator (AFG3051C, Tektronix Inc., Beaverton, Oregon, USA). For each step, the data were recorded for 1 min, and the averaged result was used to evaluate the error to the reference voltage. The crosstalk between the channels was also evaluated as the channels were converted by a common ADC. In this test, CH1 to CH4 were connected to the reference electrode (reference zero), and DC voltage was fed into CH5 ranging from −400 mV to 400 mV by the functional generator with a step of 100 mV. The sampling interval between each channel was set to a different value by modifying the counting speed of the clock interrupt in the embedded system of the MCU, which controls the switching interval of the MUX and the sample rate of the ADC.

### 2.6. Measurement of Inorganic Ions in Sample Solution 

Before measurement, the DES and SPD were assembled together tightly, and the battery in the SPD was activated to power the whole system. To clean the SC-ISEs, the iCapsuleEC was rinsed with distilled water. Then, the device was put into distilled water for 1 h. To carry out a cycle of measurement, a high-concentration salt solution was added to the solution under stirring to adjust the concentration of the target ion, and the potential responses of each SC-ISE was recorded with different concentrations of the target ion. 

## 3. Results and Discussion

### 3.1. Accuracy of the iCapsuleEC for Multichannel Measurement 

**Figure 3:**
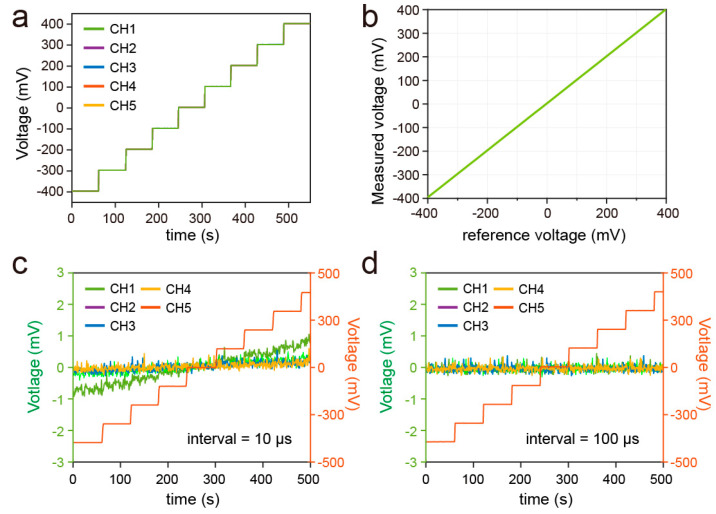
Response of the channels when connected to a standard voltage signal as it increased from −400 mV to 400 mV (**a**). Response of the channels when CH5 was connected to a stepwise signal and other channels were connected to the ground (**b**). The crosstalk between the channels with a multiplexer switching interval of 10 μs (**c**) and 100 μs (**d**). The value series on the right vertical axis corresponds to the line of CH5.

The accuracy of the SPD for multichannel measurements was first examined by connecting the channels to a common DC source. As a result, the voltage measured by the SPD for all five channels was well consistent with the input voltage, as the mean related error was less than ±0.5%. In addition, the mean standard deviation of the multichannel measurement was only 0.1056 in the range of −400 mV to 400 mV, indicating the good consistency of the channels (Figure 3a,b). As the signals of all channels were converted by a common ADC, we also examined the crosstalk between the channels by connecting one channel (CH5) to the DC source with input voltage ranging from −400 mV to 400 mV. Another channel was connected to the reference zero. The crosstalk between the channels was investigated with a switch interval of 10 μs and 100 μs. The result demonstrates that the data of CH1, which was measured after CH5, was interfered with by the signal of CH5 with a switch interval of 10 μs, while the other channels were less affected. The effect appears to be significant with the increase of the voltage difference between the two channels (Figure 3c). On the contrary, the crosstalk was not observed by increasing the switch interval to 100 μs (Figure 3d). This unwanted crosstalk was caused by the capacitance in the signal-processing circuit before the ADC, which has a discharging process after channel switching. Therefore, by increasing the interval, the rise time was not sampled by the ADC.

### 3.2. Optimization of the Solid-Contact Layer for the SC-ISEs

**Figure 4:**
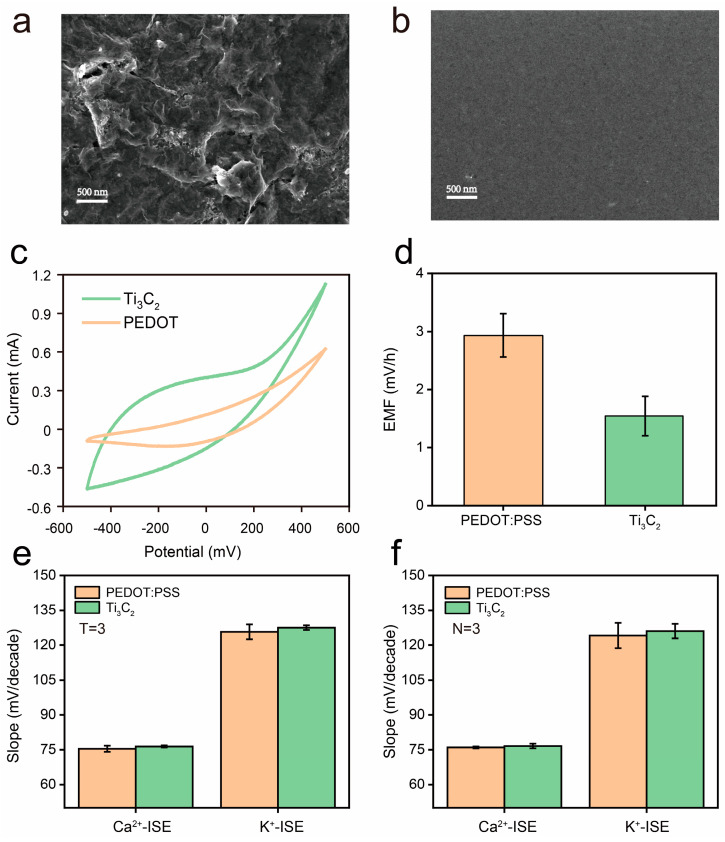
TEM photograph of the formed membrane of the Ti_3_C_2_−modified (**a**) and PEDOT:PSS−modified (**b**) Au electrodes made using the dipping method. (**c**) The cyclic voltammetry of the Ti_3_C_2_− and PEDOT:PSS−modified Au electrodes in 1 M NaCl, respectively. (**d**) The drifting of the EMF (N = 3). (**e**) Repeatability of the EMF response of the Ca^2+^ ISM and K^+^ ISM to solid contact of PEDOT:PSS and Ti_3_C_2_ (T = 3). (**f**) The consistency of the Ca^2+^ ISM and K^+^ ISM with solid contact of PEDOT:PSS and Ti_3_C_2_ (N = 3).

Generally, the solid-contact layer has significant influence on the stability of EMF, which is a challenge to eliminate. Many studies focused on the suppression of the drift through optimization of electrode materials or the development of new methods for signal readout, while more efforts are still needed to improve the performance of SC-ISEs for practical use. Here, to demonstrate the ability of the iCapsuleEC to study and optimize the electrode, Ti_3_C_2_ was introduced as an alternative material for the solid contact. Both PEDOT:PSS and Ti_3_C_2_ were prepared as solid-contact layers on the PCB-Au substrate, respectively. When characterized by field emission scanning electron microscopy, it is obvious that the surface of the PEDOT:PSS-modified PCB-Au electrode (Figure 4b) is much flatter than that of the Ti_3_C_2_-modified PCB-Au electrode (Figure 4a). In addition, the surface of the Ti_3_C_2_-modified PCB-Au electrode is uneven and rough, suggesting a larger specific surface area which increases the contact area between the Ti_3_C_2_ solid-contact layer and the ion-selective membrane, thereby increasing the double-layer capacitance [27]. Then, the electrical properties of the Ti_3_C_2_- and PEDOT:PSS-modified PCB-Au electrodes were characterized by using cyclic voltammetry (CV) in a 1 M NaCl solution at a scan rate of 100 mV/s with potential scanning from −500 mV to 500 mV (vs. Ag/AgCl) using a custom-developed electrochemical system [33]. As shown in Figure 4c, the areal specific capacitance of the Ti_3_C_2_-modified Au electrode (27.64 mF/cm^2^) is much larger than that of the PEDOT:PSS-modified PCB-Au electrode (9.98 mF/cm^2^), which is estimated by the CV loops.

In order to compare the stability of SC-ISEs based on PEDOT:PSS and Ti_3_C_2_, the potential drift of the PCB-Au/Ti_3_C_2_/Ca^2+^-ISEs and PCB-Au/PEDOT:PSS/Ca^2+^-ISEs was measured in a 10 mM CaCl_2_ solution for 2 h. As a result, the mean potential drift of the PEDOT:PSS-based SC-ISEs was 2.9 ± 0.38 mV/h, while the potential drift of the Ti_3_C_2_-based SC-ISEs was 1.5 ± 0.34 mV/h (Figure 4d). The result indicates that the Ti_3_C_2_-based ISEs can provide less drifting than the PEDOT:PSS-based SC-ISEs.

To evaluate the sensitivity of the ISEs, the potential response of the PEDOT:PSS-based SC-ISEs and Ti_3_C_2_-based SC-ISEs was measured in CaCl_2_ and KCl solutions with concentrations ranging from 0.1 μM to 23.79 mM, respectively. All the measurements were carried out by the iCapsuleEC, with five SC-ISEs measured at a time. The EMF of the Ti_3_C_2_-based ISEs and the PEDOT:PSS-based SC-ISEs was linear with the logarithm of the concentration of Ca^2+^ and K^+^ (Figure 4e,f). The slopes of the EMF responses were then evaluated (Table S1). It is worth noting that the slope of Ti_3_C_2_-based SC-ISEs appears to have better repeatability than the slope of the PEDOT:PSS-based ISEs as it has smaller deviations for multitime measurements (Figure 4e). The slope variations among different ISEs were not significant for either the Ti_3_C_2_-based SC-ISEs or the PEDOT:PSS-based SC-ISEs (Figure 4f).

According to the results, the SC-ISEs appear to have a higher slope than the Nernst slope. Similar results were also reported in other studies of SC-ISEs. The mechanism of the results could be induced by the additional interactions of the cations and the solid-contact layer of PEDOT:PSS and Ti_3_C_2_, such as cation–π interactions, and the absorbance in the interlayer spacing of the materials, leading to the accumulation of cations in the solid-contact layer [34,35]. In addition, the oxidation in the interlayer spacing of the two-dimensional Ti_3_C_2_ material could also increase the double-layer capacitance of the electrode and the response slope [36,37], as observed in the TEM image with the rod-shaped particles (Figure S1). The EMF response could also be affected by the reference electrode due to the concentration change in Cl^-^ during measurement. Since the mechanism of the solid-contact layer is not the concern of this study, the hypothesis is not verified here.

In summary, both the Ti_3_C_2_-based and PEDOT:PSS-based SC-ISEs have a stable response slope. The Ti_3_C_2_-based SC-ISEs have less signal drifting than the PEDOT:PSS-based SC-ISEs. Due to the larger capacitance of the Ti_3_C_2_ layer, the Ti_3_C_2_-based SC-ISEs can effectively convert the ion signal in the ion-selective membrane into an electronic signal, resulting in good sensitivity and repeatability.

### 3.3. Performance for Detection of K^+^, NH_4_^+^, Na^+^, Ca^2+^, and Mg^2+^

**Figure 5:**
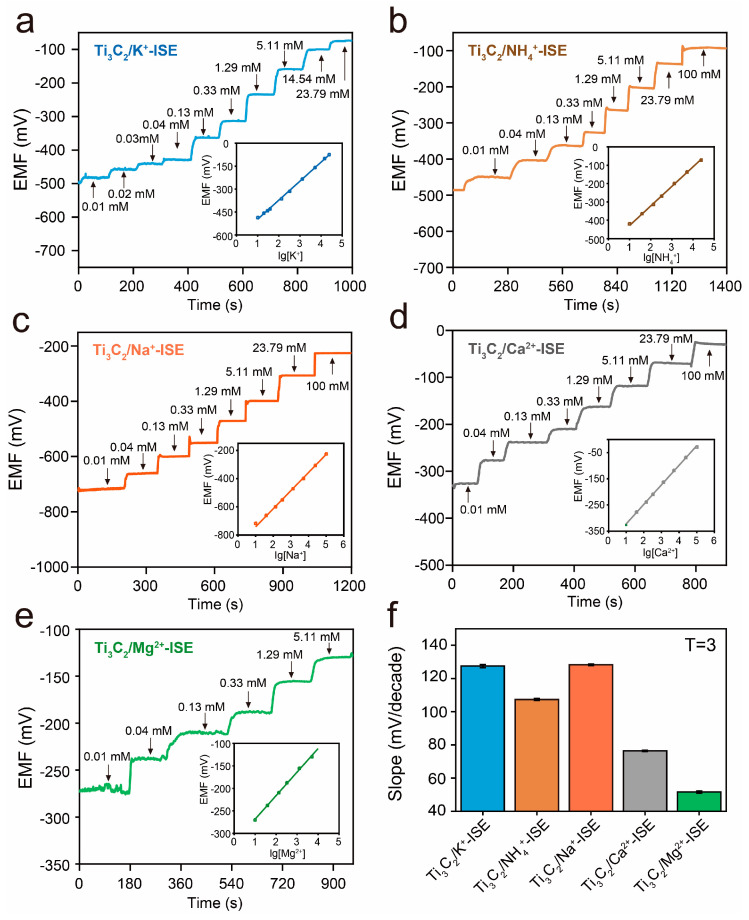
(**a**–**e**) Response of the Ti_3_C_2_/K^+^−ISE, Ti_3_C_2_/NH_4_^+^−ISE, Ti_3_C_2_/Na^+^−ISE, Ti_3_C_2_/Ca^2+^−ISE, and Ti_3_C_2_/Mg^2+^−ISE. (**f**) Response slope of the five ISEs (T = 3).

SC-ISEs with an ion-selective membrane for K^+^, NH_4_^+^, Na^+^, Ca^2+^ and Mg^2+^ were fabricated with a solid-contact layer of Ti_3_C_2_ on the DES. The potentiometric performance characteristics of each type of SC-ISE were examined in standard solutions (Figure 5a–e). After each increase in primary ion concentrations ranging from 0.01 to 100 mM, all Ti_3_C_2_-based SC-ISEs responded immediately and reached a stable potentiometric response within 20 s. As can be seen, a linear range of the developed K^+^, NH_4_^+^, Na^+^, and Ca^2+^ SC-ISEs from 0.01 to 100 mM were observed with stable response slopes (Figure 5a–d). In the case of the Mg^2+^ SC-ISE, a linear range between 0.01 and 5.11 mM of Mg^2+^ with a Nernstian slope of 51.64 mV per decade was observed (Figure 5e). The averaged slopes of the EMF response to the logarithm of the ion concentration are listed (Table S2); their corresponding standard deviations were obtained by repeating the measurements three times for each of the SC-ISEs (Figure 5f). It can be seen that all five SC-ISEs exhibit good Nernst response and potential stability under multiple long-time measurements, and the rising time of the electrodes is relatively short.

To assess the ability of Ti_3_C_2_-based SC-ISEs to distinguish and capture the target ion from potentially interfering ions, we used the separate solution method (SSM) to estimate the potentiometric selectivity of five Ti_3_C_2_-based SC-ISEs in KCl, NH_4_Cl, NaCl, CaCl_2_, and MgCl_2_ aqueous solutions with different concentrations ranging from 0.01 to 100 mM, respectively [38]. The potentiometric selectivity coefficients were calculated and are presented in Figure S2. K^+^, NH_4_^+^, Na^+^, and Ca^2+^ SC-ISEs exhibited good selectivity in most of the concentrations. The Mg^2+^ SC-ISE was affected by interferential ions in low concentrations when detected in solutions containing multi-ions, which decreased the linear range. The design of the multiplexed SC-ISEs sensor array contributes to the measurement of the interference of interferential ions quickly and efficiently in a complex mixed-solutions system. As can be seen from Figure S2, each SC-ISE had different selectivity for the measured ions in solutions containing different ions. By further introducing machine learning-based algorithms and models, it is possible to correct the interference, and the actual concentrations of primary ions can be calibrated and calculated.

### 3.4. Multichannel Analysis in Simulated Cell Culture Media

**Figure 6:**
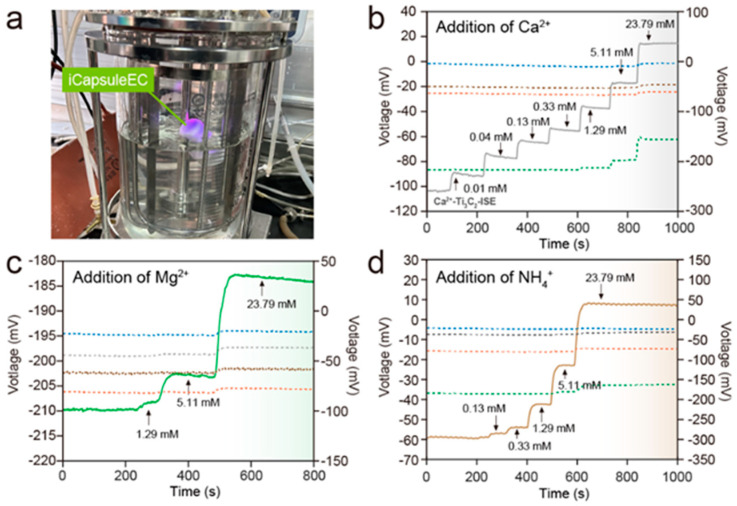
(**a**) Photograph of the iCapsuleEC loaded in a 5 L bioreactor. (**b**) Response of the five ISEs to the addition of Ca^2+^ in simulated MEM salt solution. (**c**) Response of the five ISEs to the addition of Mg^2+^ in simulated MEM salt solution. (**d**) Response of the five ISEs to the addition of NH_4_^+^ in simulated MEM salt solution. The value series on the left and right vertical axes correspond to the solid lines and dashed lines, respectively. The blue line is Ti_3_C_2_/K^+^−ISE, red line is Ti_3_C_2_/Na^+^−ISE, gray line is Ti_3_C_2_/Ca^2+^−ISE, green line is Mg^2+^−ISE, and brown line is Ti_3_C_2_/NH_4_^+^−ISE.

As tested, the iCapsuleEC can float and transfer data through BLE (Bluetooth low energy) in a 5 L bioreactor with all the SC-ISEs immersed in the solution (Figure 6a). To further evaluate its ability for monitoring the variation of ion concentrations in cell culture media, the iCapsuleEC was used in a simulated MEM (minimum essential medium) solution, which is a common cell culture medium that can be used for the cultivation of suspended and adherent mammalian cells, including HeLa, BHK-21, fibroblasts, and primary rat astrocytes. The simulated MEM was prepared based on the inorganic ions in the recipe for MEM (S3), and the experiment was carried out in a 1 L beaker with continuous stirring.

According to the MEM recipe, the concentration of Mg^2+^ and Ca^2+^ is much lower than the concentration of K^+^ and Na^+^. In addition, NH_4_^+^ is generated during cell culture, which does not exist in fresh MEM. Here, the iCapsuleEC was used to monitor the dynamic potentiometric response to the concentration change in Mg^2+^, Ca^2+^, and NH_4_^+^ in the simulated MEM with interference from K^+^ and Na^+^. A s a result, the EMF of the ISEs for K^+^, NH_4_^+^, and Na^+^ remained basically stable when the concentration of Ca^2+^ was merely increased, and the EMF of Ca^2+^-ISE increased significantly when the concentration was higher than 1 mM (Figure 6b).

Compared with the other four SC-ISEs, the EMF of the Mg^2+^-ISE in MEM solution changed significantly when the concentration of Mg^2+^ was higher than 1 mM (Figure 6c). The NH_4_^+^-ISE could well distinguish and capture the target NH_4_^+^ from the mixed solution when the concentration of NH_4_^+^ was higher than 100 µM (Figure 6d).

Although the response was reduced compared to those measured in standard solutions, the EMF of the SC-ISEs still exhibited a strong relationship with the concentration of the target ions. As the selectivity of all the SC-ISEs can be evaluated and the bioreactions are always carried out in a certain condition, the device is promising for monitoring ion concentrations by modeling the ion change with the EMF of the SC-ISEs. Therefore, methods based on machine learning can be used to recover the information of the target ions from the disturbed signals. Recently, a random forest algorithm was used for accurate ion-type classification, concentration prediction, and diagnosis of electrolyte-imbalance-related diseases based on a graphene-based bioelectronic sensing platform [39]. For further application in bioreactors, more improvements can also be made to enhance the performance of the device, such as increasing the SC-ISE number for data fusion and integrating a microfluidic system for online calibration with standard solutions.

## 4. Conclusions

This work reports on the iCapsuleEC system for in situ monitoring of inorganic ions in bioreactors. The iCapsuleEC system has advantages including small volume, multichannel detection, high integration, and wireless usage. The sensing part is designed to be a disposable electrochemical sensor with the integration of five SC-ISEs and a reference electrode. The electronics of the iCapsuleEC with a self-calibration feature can measure and process the potential signal of the five SC-ISEs and transmit the data using the BLE technique. The whole system is powered by a Li-ion battery; therefore, it can run wirelessly in a bioreactor for long-time usage. The stability test of the signal-processing device indicates a high accuracy of the device for measurements of the five channels without crosstalk at an optimized measuring interval. The solid-contact layers of the SC-ISEs were optimized by Ti_3_C_2_ for the detection of different ions, which had a lowered potential drift (1.5 mV/h) when compared with the PEDOT:PSS-based SC-ISEs. Then, the SC-ISEs with different ion-selective layers were fabricated for the detection of K^+^, NH_4_^+^, Na^+^, Ca^2+^, and Mg^2+^. The performance and selectivity of the electrodes were investigated by using the iCapsuleEC. The results show the ability of all the SC-ISEs to detect the ions with good linearity in a wide range of ion concentrations. The ability of Ti_3_C_2_-based SC-ISEs to distinguish and capture the target ion from potentially interfering ions was assessed with the separate solution method (SSM), and all SC-ISEs exhibited high selectivity to target ions even with the existence of the other four interferential ions. Finally, the iCapsuleEC was tested in a simulated MEM salt solution to check the possibility for evaluating changes in ions in cell culture media. The results revealed that the integrated SC-ISEs can estimate changes in ion concentration even when they are interrupted by other inorganic ions. Although the EMF of the SC-ISEs was not linear to the ion concentration in the simulated MEM solution due to the existence of interfering ions, which is consistent with the Nicolsky–Eisenman equation, the iCapsuleEC provided a data basis for the collection of the relationships between the solution content and the change in the EMF value. To further enhance the performance of the proposed sensor system in real applications, more effort is necessary to modify the design of the reference electrode to increase its potential stability in a dynamic environment. In the future, the processing of the sensor data can be further improved by introducing machine learning approaches based on the large data set obtained by the sensor networking with the iCapsuleEC system.

## Figures and Tables

**Table 1:** Composition of ion-selective membrane cocktails used for ISEs.

ISE	Ionophore	Ionic Additive	Plasticizer	Main Solid Component	Reference
K^+^	1.00 wt% valinomycin	0.40 wt% Na-TFPB	65.70 wt% DOS	32.90 wt% PVC	[28]
NH_4_^+^	1.00 wt% nonactin	0.50 wt% KTpClPB	33.00 wt% DOS	65.50 wt% PVC	[29]
Na^+^	1.00 wt% Na ionophore X	0.55 wt% Na-TFPB	65.45 wt% DOS	33.00 wt% PVC	[30]
Ca^2+^	1.00 wt% ETH 5324	0.50 wt% Na-TFPB	65.50 wt% DOS	33.00 wt% PVC	[31]
Mg^2+^	1.40 wt% Mg ionophore I	1.00 wt% KTpClPB	64.50 wt% 2-NPOE	33.10 wt% PVC	[32]

## Data Availability

The data that support the findings of this study are available from the corresponding author upon reasonable request.

## Supplementary Materials

The following supporting information can be downloaded at: https://www.mdpi.com/article/10.3390/bios13100914/s1, S1. Chemicals and materials; Figure S1: (a–c) TEM photograph of Ti_3_C_2_; (d) AFM photograph of Ti_3_C_2_; (e) the thickness data for Ti_3_C_2_ at the white line in (d); Figure S2: (a–e) Selectivity coefficients of the five Ti_3_C_2_-based ISEs for four other ions at different concentrations; (f) response slopes of the five Ti_3_C_2_-based ISEs; Table S1: The response slope of PEDOT:PSS-based and Ti_3_C_2_-based SC-ISEs; Table S2: The response slope of the five Ti_3_C_2_-based SC-ISEs; Table S3: The recipe of MEM.
