# Supplementary material for: A Floating Capsule Electrochemical System for In Situ and Multichannel Ion-Selective Sensing
Source: Biosensors (Basel). 2023 Oct 5;13(10):914. doi: 10.3390/bios13100914 (PMC10605769; doi:10.3390/bios13100914)
Supplement: Supplementary file 1 [file biosensors-13-00914-s001.zip › biosensors-2621609-supplementary.pdf]

S1. Chemicals and Materials

Na ionophore X, Mg ionophore I, and Ca ionophore IV (ETH 5324) were obtained from Sigma-Aldrich Inc. (USA). Nonactin, 2-nitrophenyl octyl ether (2-NPOE), and tetrahydrofuran were obtained from Shanghai Aladdin Bio-Chem Technology Co., Ltd. (Shanghai, China). Bis(2-ethylhexyl) sebacate (DOS) was obtained from TCl (Shanghai) Co., Ltd. (Shanghai, China). Potassium tetrakis(4-chlorophenyl)borate (KTpClPB) was obtained from Alfa Aesar China Chemical Co., Ltd. (Shanghai, China). Valinomycin, sodium tetrakis[3,5-bis(trifluoromethyl)phenyl]borate (Na-TFPB), polyvinyl chloride (PVC, K-value: 68–65), sodium chloride (NaCl), potassium chloride (KCl), magnesium chloride ($MgCl_2$), calcium chloride ($CaCl_2$), ammonium chloride (NH_4Cl), magnesium sulfate ($MgSO_4$), sodium bicarbonate ($NaHCO_3$), and sodium dihydrogen phosphate (NaH_2PO_4) were obtained from Shanghai Macklin Biochemical Technology Co., Ltd. (Shanghai, China). Ti_3C_2 (5 mg/ml) was obtained from Shandong Xiyan New Material Technology Co., Ltd. (Shandong, China). Poly(3,4-ethylenedioxythiophene) polystyrene sulfonate (PEDOT:PSS) conducting polymer solution was obtained from Beijing J&K Scientific Co., Ltd. (Beijing, China). PDMS (Sylgard 184) was obtained from Dow Corning, Inc. (USA) to seal the electronic items. All solutions were prepared using distilled water purchased from Watsons (Shanghai, China).

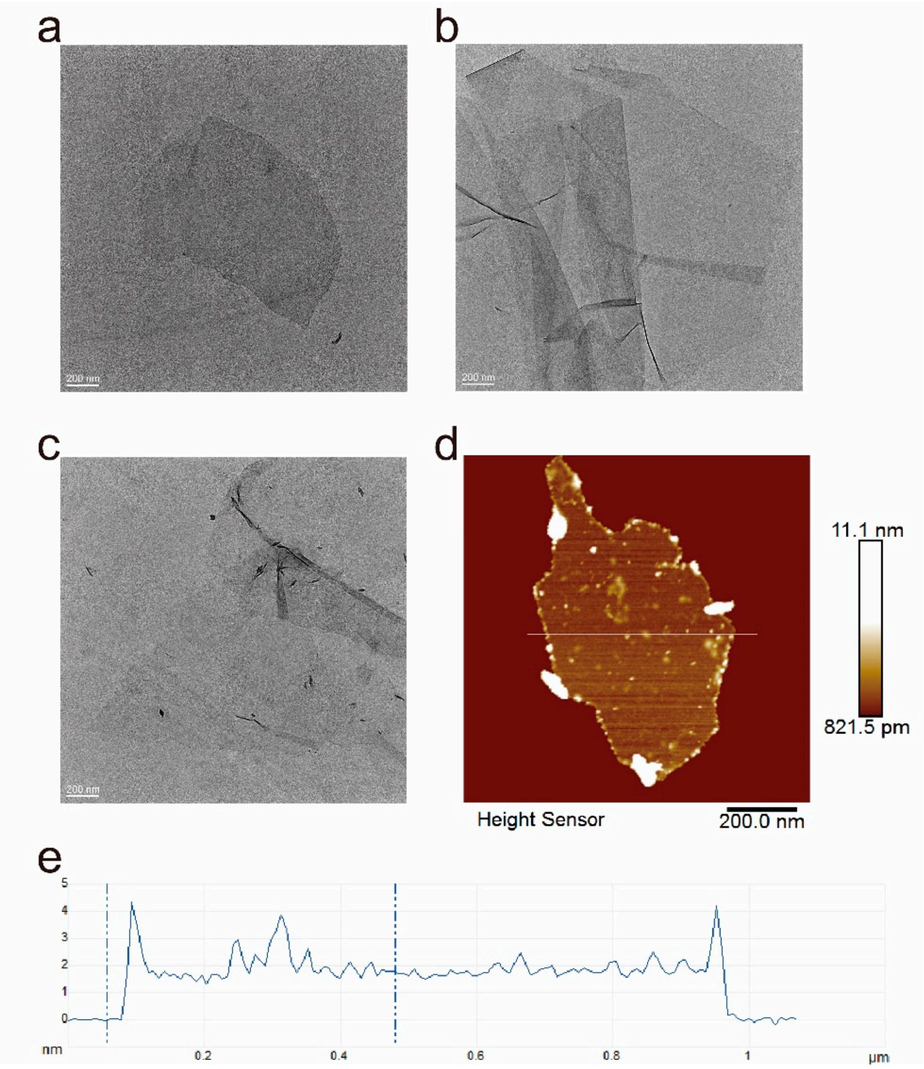

Figure S1. (a-c) TEM photograph of Ti_3C_2; (d) AFM photograph of Ti_3C_2; (e) the thickness data for Ti_3C_2 at the white line in (d).

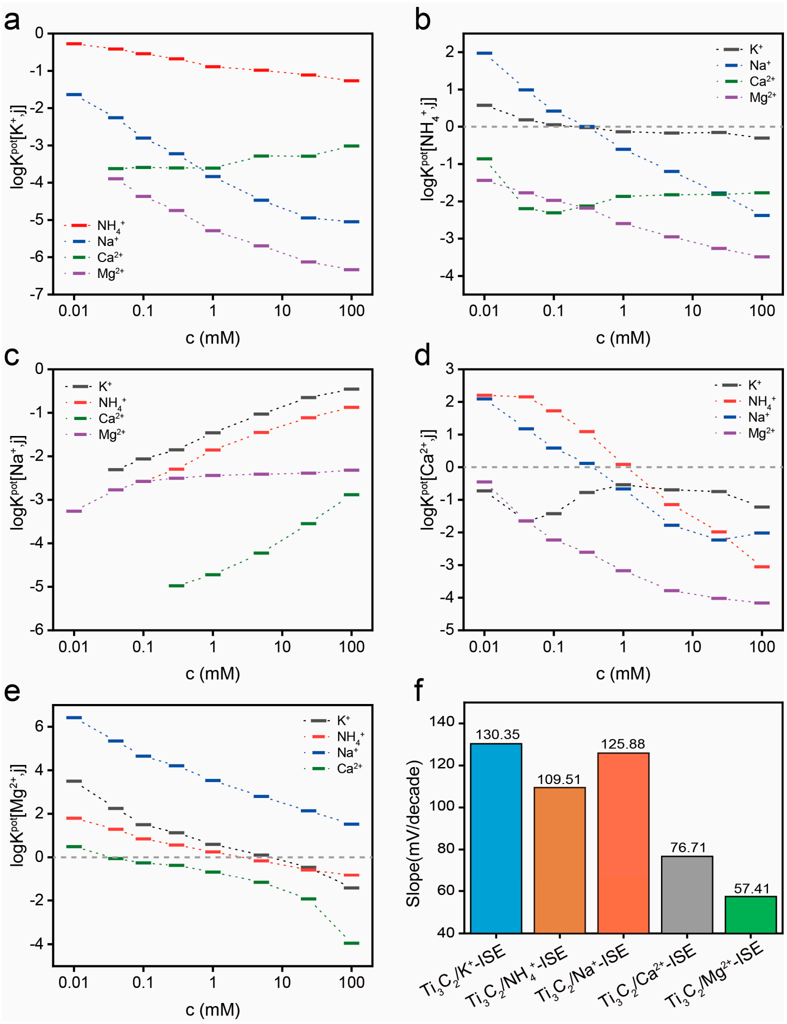

Figure S2. (a-e) Selectivity coefficients of the five Ti_3C_2-based ISEs for four other ions at different concentrations; (f) response slopes of the five Ti_3C_2-based ISEs.

Table S1. The response slope of PEDOT:PSS-based and Ti_3C_2-based SC-ISEs.

	SC-ISE	Response Slope (mV/decade)
T=3	PCB-Au/PEDOT:PSS/Ca^{2+}-ISE	75.42 ± 1.32
	PCB-Au/Ti_3C_2/Ca^{2+}-ISE	76.44 ± 0.45
	PCB-Au/PEDOT:PSS/K^+-ISE	125.69 ± 3.18
	PCB-Au/Ti_3C_2/K^+-ISE	127.49 ± 0.99
N=3	PCB-Au/PEDOT:PSS/Ca^{2+}-ISE	76.02 ± 0.42
	PCB-Au/Ti_3C_2/Ca^{2+}-ISE	76.64 ± 0.99
	PCB-Au/PEDOT:PSS/K^+-ISE	124.13 ± 5.45
	PCB-Au/Ti_3C_2/K^+-ISE	126.03 ± 3.11

Table S2. The response slope of the five Ti_3C_2-based SC-ISEs.

SC-ISE	Response Slope (mV/decade)	R^2
PCB-Au/ Ti_3C_2/K^+-ISE	115.48 ± 0.99	0.9954
PCB-Au/ Ti_3C_2/NH_4^+-ISE	96.82 ± 0.57	0.9937

PCB-Au/ Ti_3C_2/Na^+-ISE	128.25 ± 0.44	0.9994
PCB-Au/ Ti_3C_2/Ca^{2+}-ISE	76.44 ± 0.45	0.9993
PCB-Au/Ti_3C_2/Mg^{2+}-ISE	51.64 ± 0.63	0.9984

Table S3. The recipe of MEM.

Inorganic Salts	Molecular Weight	Concentration (mg/L)	mM
Calcium chloride ($CaCl_2$)	111.0	200.0	1.8
Magnesium sulfate ($MgSO_4$)	120.0	97.67	0.8
Potassium chloride (KCl)	75.0	400.0	5.3
Sodium bicarbonate ($NaHCO_3$)	84.0	2200.0	26.2
Sodium chloride (NaCl)	58.0	6800.0	117.2
Sodium phosphate monobasic (NaH_2PO_4-H_2O)	138.0	140.0	1.0
